# Fetal Cerebral Hemodynamic Changes in Preeclampsia Patients by Ultrasonic Imaging under Intelligent Algorithm

**DOI:** 10.1155/2022/4269308

**Published:** 2022-05-27

**Authors:** Di Zhu, Ru Ding, Hongxia Ma, Shenglin Jiang, Lijie Li

**Affiliations:** ^1^Department of Obstetrics, Affiliated Zhejiang Hospital, Zhejiang University School of Medicine, Hangzhou 310030, Zhejiang, China; ^2^Department of Gastroenterology, Shulan (Hangzhou) Hospital Affiliated to Zhejiang Shuren University Shulan International Medical College, Hangzhou 310022, Zhejiang, China; ^3^Department of Ultrasound Medicine, Affiliated Zhejiang Hospital, Zhejiang University School of Medicine, Hangzhou 310030, Zhejiang, China

## Abstract

This study was aimed at evaluating the adoption value of ultrasound imaging features on fetal cerebral hemodynamics in preeclampsia patients based on the partial difference algorithm and the hybrid segmentation network (HSegNet) algorithm. Forty pregnant women with preeclampsia diagnosed by ultrasound examination were selected as the research objects, and another forty normal pregnant women were selected as the control. Then, by using the partial differential algorithm, the imaging of fetal cerebral hemodynamics in preeclampsia patients was enhanced and optimized, and the general clinical data and experimental results were collected. The results showed that the automatic labeling of fetal cerebral artery in fetal middle cerebral artery (MCA) hemodynamic images was realized by HSegNet algorithm model, and the final accuracy was 97.3%, which had a good consistency with the manual annotation of doctors. Education level was a protective factor for preeclampsia (odds ratio (OR) = 0.535). Body mass index (BMI) and family history of hypertension during pregnancy were independent risk factors for preeclampsia (OR = 1.286, and 2.774, respectively). MCA end-diastolic volume (EDV) of preeclampsia fetuses was higher than that of normal fetuses. The MCA systolic-diastolic ratio (S/D), the pulsatility index (PI), and the resistive index (RI) in the preeclampsia group were significantly lower than those in the normal pregnancy group. The results showed that MCA PI, MCA RI, and MCA *S*/*D* had certain predictive values for the occurrence of adverse pregnancy outcomes (*P* < 0.05). In summary, the intelligent algorithm-based fetal MCA hemodynamic ultrasound image in the study could effectively predict pregnancy outcomes of patients and provide certain theoretical support for the subsequent reduction of adverse pregnancy outcomes in patients with preeclampsia.

## 1. Introduction

Eclampsia is a major complication of pregnancy that affects about 4% to 5% of pregnancies worldwide. The disease is one of the leading causes of maternal and fetal morbidity and mortality, as well as an important cause of premature birth and long-term cardiovascular disease in mothers. Eclampsia is defined as emerging hypertension and proteinuria or other terminal organ damage after 20 weeks of gestation [1, 2]. Some scholars have pointed out that epileptic patients have a high risk of seizures, and the risk of epileptic seizures in patients with early eclampsia will be further increased [3, 4]. Other researchers have found that impaired placental implantation in eclampsia patients may lead to extensive endothelial dysfunction [5]. At present, American College of Obstetricians and Gynecologists Working Group divided gestational hypertension into four categories: preeclampsia, chronic hypertension, chronic hypertension with preeclampsia, and gestational hypertension [5].

Patients with any of the following severe features are usually diagnosed as having preeclampsia: (I) patients with systolic blood pressure ≥160 mmHg or diastolic blood pressure ≥110 mmHg; (II) patients with the platelet count <100 × 109/L; (III) patients with abnormally elevated liver enzymes to twice normal concentrations or severe persistent right epigastric or epigastric pain as the evidence of impaired liver function; (IV) patients with renal failure and the serum creatinine level >1.1 mg/dl or the double serum creatinine levels; (V) patients with pulmonary edema, cerebral infarction at acute stage, or visual impairment [6, 7]. Low molecular weight heparin was first applied in the prevention and treatment of venous thromboembolism and pulmonary embolism in pregnant women, and it has certain safety for both mothers and infants. In recent years, it has been widely used to prevent preeclampsia in pregnant women who are prone to thrombosis. Clinically, for the patients with high blood coagulation tendency or a history of preeclampsia, using the low molecular weight heparin again during pregnancy can effectively reduce the recurrence of preeclampsia or improve pregnancy outcomes [8]. Traditional treatment for early-onset eclampsia, especially severe preeclampsia, mostly advocates immediate termination of pregnancy regardless of gestational age. However, most fetuses are immature, which results in perinatal death [9]. In recent years, with the continuous development of obstetric intensive medical monitoring, nursing technology, and neonatal intensive care technology, the treatment of early-onset preeclampsia has also been obviously improved. Expectant management is a relatively common method for the treatment of early-onset eclampsia, and it has received widespread clinical attention in terms of the improvement of pregnancy outcomes and the safety of mother and infant [10].

Ultrasound examination is one of the most widely used imaging examination methods in the clinic and has high specificity for many gynecological diseases. Its advantages include simple operation, noninvasive, and low examination cost [7, 11], so it is generally accepted by patients. However, in the specific processes of examination imaging, there will be blur, artifact, noise, and other phenomena in the image data due to limb movements, special examination sites, heartbeats, and respiration. However, damaged images can be repaired well by image restoration methods on account of the partial differential algorithm [12], so that images can be restored to contain more rich image feature information. Fetal brains are relatively sensitive to intrauterine hypoxia. Middle cerebral artery (MCA) Doppler hemodynamic parameters are detected by color ultrasonic diagnostic instruments to judge intrauterine hypoxia. The decrease of MCA and blood spectrum resistance parameters in preeclampsia fetuses is a manifestation of compensatory dilation of MCA, which increases brain blood flow to ensure the blood supply to fetal brains. This is called the “brain protection effect.” Nevertheless, the image cutting algorithm of traditional ultrasound has poor definition and low resolution for showing tissue and lesion site. To improve image quality, in recent years, intelligent optimization algorithms have been applied to the field of medical images, such as image recognition and image segmentation, which have greatly improved image quality [13].

To sum up, the intelligent algorithm based on the partial difference algorithm and HSegNet algorithm was adopted to improve the ultrasound images, the changes of fetal cerebral hemodynamics in patients with preeclampsia were evaluated, and its application effect was explored. It was hoped to provide data and theoretical support for future clinical diagnosis of fetal cerebral hemodynamics in patients with preeclampsia.

## 2. Methods

### 2.1. Study Objects

Forty pregnant women with preeclampsia and forty pregnant women with normal pregnancies diagnosed by ultrasound examinations in the hospital were selected as the subjects. General clinical data such as education levels, history of hypertension, and imaging findings were collected. The age range was between 24 and 45 years old, with an average age of 36.4 ± 2.3 years. All patients did not have other intrauterine diseases such as intrauterine polyps and submucosal fibroids. The multicenter screening study involved fetal cerebral artery Doppler ultrasound at 22 to 37 weeks of gestation in single-pregnancy women receiving routine prenatal care. All the patients and their families understood the study and signed informed consent. The study had been approved by the ethics committees of the hospital.

Inclusion criteria were as follows. First, patients' clinical symptoms met the diagnostic criteria for preeclampsia. Second, no other pregnancy complications were identified during routine clinical examinations. Third, pregnant women received clinical prenatal examinations and had no other hidden dangers. Fourth, patients signed informed consent forms for treatments.

Exclusion criteria were as follows. First, patients did not meet the clinical diagnostic criteria for preeclampsia. Second, patients had other pregnancy complications during routine clinical examinations. Third, patients could not have ultrasound for other reasons. Fourth, patients did not sign informed consent for treatments.

### 2.2. Preeclampsia and Ultrasound Fetal Protocols

The ultrasound instrument was used in this study. For a transvaginal scan, a woman was asked to empty her bladder and place the ultrasound instrument in a stone position on her back. An ultrasound probe was inserted into the vagina and placed in the anterior fornix to measure cervix length. The probe was inserted into the lateral fornix and the color Doppler was used to identify the uterine artery at the cervical level. Three similar continuous waveforms were obtained using pulsed wave Doppler. The pulsatility index (PI) was measured to see if there were early diastolic notches. The same procedure was then repeated for the contralateral uterine arteries and the mean PIs of two vessels were calculated. Growth scans, blood pressure measurements, and urine protein analyses were performed at 28, 32, and 36 weeks. Women with normal uterine artery Doppler received routine prenatal care. Basic biological indicators such as biparietal diameter, head circumference, abdominal circumference, and femoral length of fetuses in patients with preeclampsia were routinely checked. In addition, it was necessary to evaluate the weight of the fetus, the status of the placenta, and the status of amniotic fluid. Fetal MCA blood flow spectrum was detected by color Doppler function.

Preeclampsia was defined according to guidelines from the International Society for the Study of Gestational Hypertension. This required two diastolic blood pressure records ≥90 mmHg in women with previously normal blood pressure, at least 4 hours apart, 300 mg or more proteinuria within 24 hours, or at least two ++ readings in dippaper analyses of midstream or catheter urine samples in the absence of a 24-hour collection.

### 2.3. Observation Indicators

Eighty patients were included in the study. Forty pregnant women with preeclampsia and forty pregnant women with normal pregnancies were diagnosed by ultrasound and were divided into the normal group and the preeclampsia group. The clinical data of puerpera, history of hypertension, imaging examination results, educational levels, pregnancies, deliveries, and body mass index (BMI) before pregnancies were collected. The relationship between these indicators and preeclampsia was analyzed. Peak systolic velocity (PSV), end-diastolic volume (EDV), PI, resistive index (RI), and systolic/diastolic (*S*/*D*) of fetal MCA were compared between the two groups. The incidences of adverse pregnancy outcomes in the two groups were followed up.

### 2.4. Repair of Transvaginal Ultrasound Images by the Differential Equation Algorithm

The integral variational image repair model (TV model) in the differential equation algorithm had relatively excellent anisotropic diffusivity, which could be used for image repairing and denoising at the same time, and was relatively simple in calculation and solution.

If the image to be repaired was *F* = *R*∪*E*, the expression of its cost function was

(1) $$\begin{array}{c} T\left(f\right)=\int_{f}r\left(\left|\nabla_{f}\right|\right)\text{d}x\text{d}y\text{.} \end{array}$$

There was an impulse function ∇_*f*_ at the edge of the area to be repaired, and the standby function should meet the following requirements. Then, the standby function was required to satisfy

(2) $$\begin{array}{c} \int_{f}\text{r}\left(\left|\nabla_{f}\right|\right)\text{d}x\text{d}y<\infty . \end{array}$$

In the differential equation algorithm model, *r*(|∇_*f*_|)  =  |∇_*f*_|, the function was

(3) $$\begin{array}{c} T\left(f\right)=\int_{f}\left|\nabla_{f}\right|\text{d}x\text{d}y\text{.} \end{array}$$

If denoising was carried out at the same time during image restoration, the following conditions should be met:

(4) $$\begin{array}{c} \delta^{2}=\frac{1}{s{\left(E\right)}_{E}}\int_{E}{\left|\text{f}-{\text{f}}_{0}\right|}^{2}\text{d}x\text{d}y, \end{array}$$

where *δ*^2^ represented the variance of Gaussian noise, *s*(*E*) represented the undamaged part of the image, *f* represented the repaired figure, and *f*_0_ represented the noise of the original image. The overall energy generic function could be obtained by combining equations (3) and (4), namely,

(5) $$\begin{array}{c} G_{\lambda }\left(f\right)=\int_{f}\left|\nabla_{f}\right|\text{d}x\text{d}y+\frac{\lambda }{2}\int_{E}{\left|f-f_{0}\right|}^{2}\text{d}x\text{d}y\text{,} \end{array}$$

where *λ* represented the Lagrange multiplier. In equation (6), the minimum value of the energy generic function of this model was then obtained.

(6) $$\begin{array}{c} E\left(f\right)=\int_{\text{w}}D\left(x,y,f,\frac{\varphi_{f}}{\varphi x},\frac{\Phi_{f}}{\varphi y}\right)\text{d}x\text{d}y\text{.} \end{array}$$

To obtain the minimum value *E*(*f*), the following requirements should be satisfied, which was shown in the following equation:

(7) $$\begin{array}{c} D_{f}-\frac{\varphi }{\varphi JC}D_{f_{x}}-\frac{\phi }{\varphi y}D_{f_{y}}=0. \end{array}$$

In the TV model of the differential equation algorithm:

(8) $$\begin{array}{c} D\left(x,y,f,\frac{\varphi_{x}}{\varphi n},\frac{\varphi_{y}}{\varphi n}\right)=\sqrt{{\left(\frac{\varphi_{f}}{\varphi_{x}}\right)}^{2}+{\left(\frac{\varphi f}{\varphi y}\right)}^{2}}\frac{\lambda }{2}{\left(f-f_{0}\right)}^{2}. \end{array}$$

Equations (8) and (7) were combined to obtain the minimum energy functional equation of TV model:

(9) $$\begin{array}{c} -\nabla \cdot \left(\frac{\nabla_{f}}{\left|\nabla_{f}\right|}\right)+\lambda \left(f-f_{0}\right)=0. \end{array}$$

For a certain point *z*=(*x*, *y*) ∈ *ω* on the image, the Lagrange multiplier in equation (9) satisfied

(10) $$\begin{array}{c} \lambda =\left\{\left\{\begin{array}{c} \begin{array}{c} \lambda z\in E \end{array}\\ Oz\in D \end{array}\right)\right). \end{array}$$

### 2.5. Labeling of Intrauterine Adhesions in Transvaginal Ultrasound Images by HSegNet Algorithm

**Figure 1:**
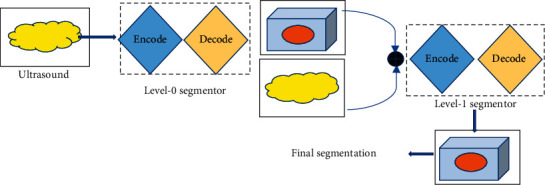
The overall framework of HSegNet algorithm.

In the study, HSegNet algorithm was used to judge and label the sites of intrauterine adhesion in ultrasound images. HSegNet algorithm which was on account of deep learning framework consisted of two-level networks and utilized automatic context models [14].  Figure 1 shows the overall framework.

HSegNet algorithm took transvaginal three-dimensional (3D) ultrasound images as input, and the encoder part of network models was used to encode it, so as to obtain high-dimensional features. Then, in the decoder part, existing doctor markers were used for model learning. HSegNet algorithm was a typical deep learning convolutional neural network. In HSegNet algorithm, it was necessary to correct the error value obtained in the calculation and use the cross-entropy loss function softmax to correct. *F*_*x*_*i*__ was set as the output *x*_*i*_ after the loss function, and then the formula of the cross-entropy loss function was

(11) $$\begin{array}{c} F_{x_{i}}=\frac{e^{x_{i}}}{\sum_{c=1}^{a}ex_{i}}. \end{array}$$

After the calculation of the cross-entropy loss function, the cross entropy of each pixel was further calculated as shown in the following equation:

(12) $$\begin{array}{c} T\left(i\right)=\sum_{a=1}^{a}y_{i,a}\log\left(d_{i},a\right), \end{array}$$

where *T*(*i*) was the cross entropy of the *i*^*th*^ pixel; *y*_*i*,*a*_ represented the true annotation of the *i*^*th*^ pixel; and *d*_*i*_, *a* represented the probability of the *i*^*th*^ pixel. The final loss cost function was shown in the following equation:

(13) $$\begin{array}{c} \text{Loss}=\frac{1}{E\times F\times p}\sum_{i=1}^{E\times F\times P}T\left(i\right). \end{array}$$

### 2.6. Statistical Standards

SPSS19.0 software was used for statistical analyses. The measurement data conforming to normal distribution were expressed as mean ± standard deviation, and differences between groups were analyzed by independent sample *t*-test. The measurement data which did not conform to normal distribution were represented by median value and quad position. The comparison between groups was analyzed by nonparametric rank sum tests. *N* (%) was used to represent the count data, and chi-square test was used to analyze differences between groups. *P* < 0.05 was considered statistically significant.

## 3. Results

### 3.1. Repair Effects of Differential Equation Algorithm on Fetal Ultrasound MAC Blood Flow Images

**Figure 2:**
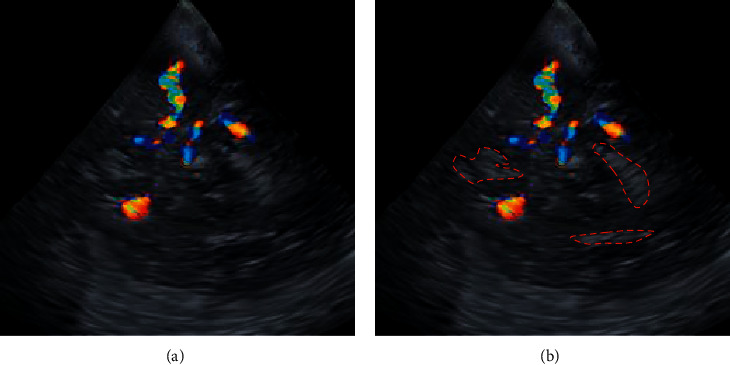
Repair of fetal ultrasound MAC blood flow images by differential equation models.

The selected fetal ultrasound MAC blood flow under-sampled images were reconstructed and optimized by image repair algorithm of differential equation models on account of deep learning. The ultrasound MAC blood flow images of fetuses were obtained with noise and blur corrected. From the comparative experimental results in Figure 2, obvious noises and blurring were contained in the under-sampled transvaginal 3D ultrasound images, and some structural images exhibited obvious blurring. After the differential equation algorithm model reconstruction, the fetal ultrasound MAC blood flow images with fewer noise and clearer structures were obtained.

### 3.2. The Effectiveness of HSegNet Algorithm Model in Fetal Ultrasound MAC Blood Flow Image Labeling

**Figure 3:**
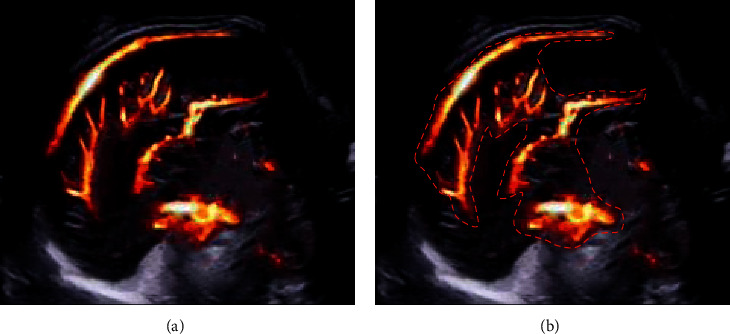
Schematic diagram of automatic labeling of HSegNet algorithm model. (a) Unmarked image. (b) Arterial blood flow in fetal ultrasound MAC blood flow image automatically labeled by the algorithm.

**Figure 4:**
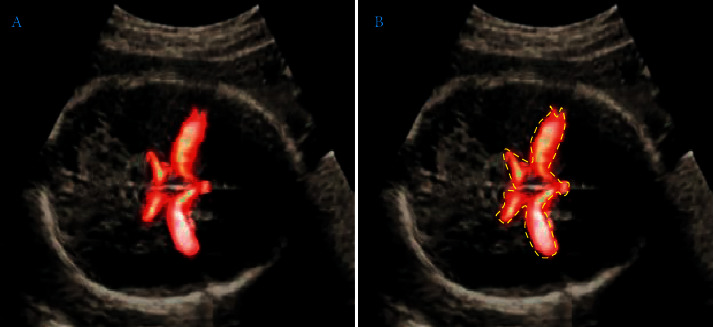
Comparison of HSegNet algorithm model between automatic annotation and manual annotation. (a) Manual annotated image by doctors. (b) Automatic annotated image by algorithm.

In the study, the HSegNet algorithm model was used to realize automatic labeling of arterial blood flow in fetal ultrasound MAC blood flow images. It was aimed to solve serious problems of clutter interference shadow in ultrasonic images. The image data were repaired and enhanced through the differential equation algorithm in previous studies. Finally, automatic context models were used to improve the accuracy of the final automatic tagging, and the automatic tagging HSegNet algorithm was trained through qualitative and quantitative analyses of experiments. The final accuracy coefficient was 97.3%, which was consistent with the manual annotation by doctors. The schematic diagram of automatic labeling is shown in Figure 3, and the comparison of manual labeling with doctors is shown in Figure 4.

### 3.3. Influencing Factors of Preeclampsia

**Figure 5:**
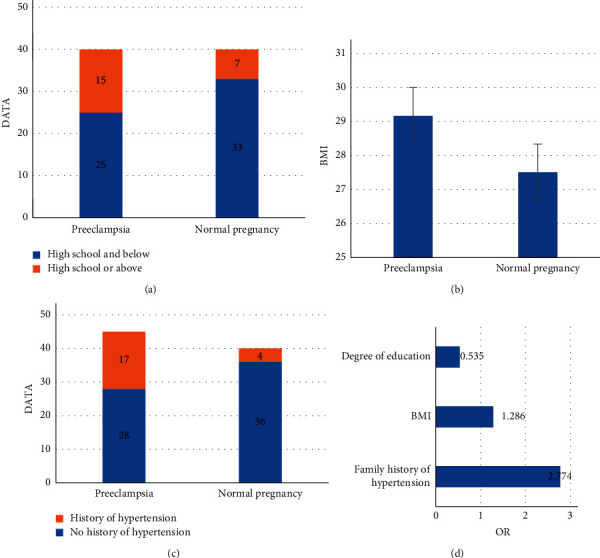
Influencing factors of preeclampsia.

As shown in Figure 5, the relationship between these indicators and preeclampsia after collecting the subjects' educational levels, history of hypertension, imaging examination results, numbers of pregnancies and births, prepregnancy BMI, and other clinical data was analyzed. As shown in Figure 5(d), preeclampsia had a certain correlation with BMI, family history of hypertension, and educational levels of pregnant women. Education level was a protective factor for preeclampsia (odds ratio (OR) = 0.535). BMI and family history of hypertension were independent risk factors for preeclampsia (OR = 1.286 and 2.774, respectively).

### 3.4. Hemodynamic Parameters of MCA in Preeclampsia Fetuses

**Figure 6:**
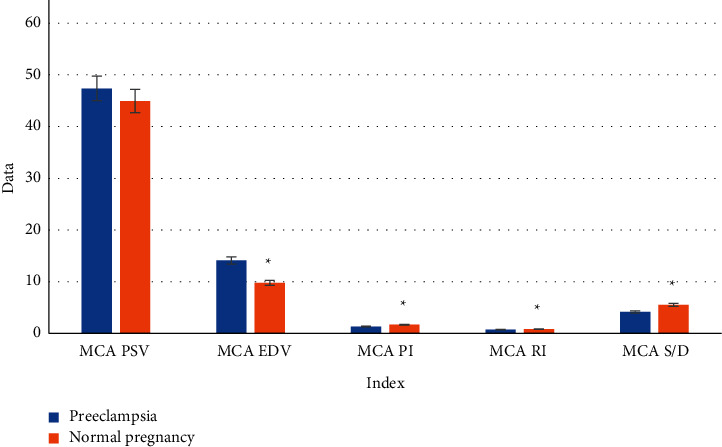
Hemodynamic parameters of MCA in preeclampsia fetuses. ∗ meant that compared with the normal fetuses, *P* < 0.05.

Fetal MCA hemodynamic tests were performed on 80 patients in the study. Various MCA hemodynamic parameters of fetuses in the preeclampsia group and the normal pregnancy group were obtained. The data of fetal MCA PSV, MCA EDV, MCA PI, MCA RI, and MCA *S*/*D* were compared between the two groups. As shown in Figure 6, MCA EDV of the preeclampsia fetuses was higher than that of the normal fetuses, and MCA *S*/*D*, PI, and RI of the preeclampsia fetuses were significantly lower than those of the normal pregnancy fetuses, with statistically significant differences (*P* < 0.05).

### 3.5. Predictive Value of MCA Hemodynamic Parameters in Preeclampsia Fetuses for Adverse Pregnancy Outcomes

**Figure 7:**
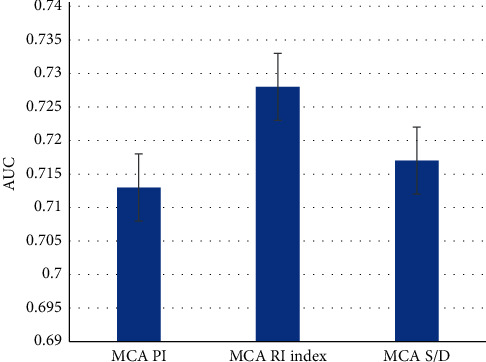
Predictive value of fetal MCA hemodynamic parameters for pregnancy outcomes.

**Figure 8:**
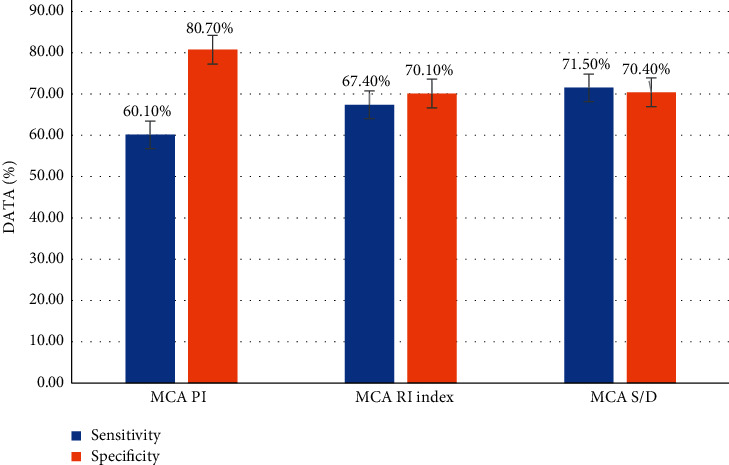
Sensitivity of fetal MCA hemodynamic parameters to predict adverse pregnancy outcomes.

According to various indexes of fetal MCA hemodynamic parameters, receiver operating characteristic curves were made. The value of these indexes in predicting adverse pregnancy outcomes was analyzed. As shown in Figures 7 and 8, MCA PI, MCA RI, and MCA *S*/*D* in the analysis results all had certain predictive values for the occurrence of adverse pregnancy outcomes (*P* < 0.05).

## 4. Discussion

Preeclampsia and eclampsia can cause more than 50,000 maternal deaths worldwide each year, and frequencies vary widely across geographical regions. The incidences of hypertension during pregnancy have increased in industrialized countries. African American women have higher related mortality rates than Hispanics, American Indians, whites, Asians, or Pacific Islander women. In contrast, the incidences of eclampsia have declined with more extensive prenatal care and the use of magnesium sulfate. According to the national hospital discharge survey, the prevalence of hypertensive disorders in pregnancy (preeclampsia, eclampsia, gestational hypertension, and chronic hypertension) was estimated at 5.9% in the United States. The survey monitored about 39 million newborns over a 10-year period [15, 16]. The study also showed that women with preeclampsia or eclampsia had a 3-25-fold increased risk of serious complications during their index pregnancies, including placental abruption, disseminated intravascular coagulation, pulmonary edema, and aspiration pneumonia [17].

Because of the differences in epidemiology, clinical presentation, and associated morbidity between early or “placental” preeclampsia (which occurs before 34 weeks) and late or “maternal” preeclampsia (which occurs after 34 weeks). The debate about the heterogeneity of preeclampsia is ongoing. For example, early onset of preeclampsia is associated with a significant risk of intrauterine growth restriction, while the late-onset disease is usually associated with maternal obesity and gestational age neonatal weight gain [18, 19]. The clinical manifestations of early and late onset of preeclampsia are different. However, transcriptional profiling studies have shown that there are common genetic signatures in the blood of mothers of both subtypes. This suggests that the mechanisms of vascular damage in mothers may be more similar than previously thought.

Determinants of preeclampsia include family histories, genetic predisposition, duration of sexual cohabitation, maternal smoking, number of pregnancies, maternal ages, use of in vitro fertilization, and maternal medical conditions such as previous family history of hypertension, diabetes, chronic kidney disease, and obesity. Conditions associated with increased placental mass such as multiple pregnancies and hydatidiform moles are also associated with an increased risk of preeclampsia, while trisomy 21 is also associated with a higher risk of preeclampsia. Pregnant women with or giving birth to preeclampsia are at increased risk during pregnancies and continue to be at increased risks after their first pregnancies. The heritability of preeclampsia is estimated at 55%, with both maternal and fetal genetic factors increasing the risk of pregnancy (30 to 35% and 20%, respectively) [20, 21]. Compelling evidence was reported by a large genome-wide association study. It showed that changes near the fms-like tyrosine kinase 1 (FLT1) site in the human fetal genome could contribute to preeclampsia.

However, in terms of fetal Doppler ultrasonography, the protective effect of fetal brains is characterized by the increased end-diastolic flow rate of MAC, which is manifested by decreased PI, RI, and *S*/*D*. The results of this experiment showed that MCA EDV of the preeclampsia group was higher than that of the normal pregnancy group, and MCA *S*/*D*, MCA PI, and MCA RI were lower than those of the normal pregnancy group. Education level was a protective factor for preeclampsia (OR = 0.535). BMI and family history of hypertension in pregnant women were independent risk factors for preeclampsia (OR = 1.286 and 2.774, respectively). The differences between groups were statistically significant (*P* < 0.001). It suggested that in preeclampsia, trophoblast cells in the spiral arteries of the mother's uterus had trouble recasting. The resistances to maternal-placental and placental-fetal circulation were increased. A lack of blood supply could cause fetal ischemia and hypoxia and the immediate redistribution of fetal blood. Therefore, solving the oxygen shortage could guarantee the blood supply of the heart, brain, and other important organs.

## 5. Conclusion

The objective of the study was to optimize and label fetal MCA hemodynamic ultrasound images by using deep learning technology and to further evaluate the prediction of adverse outcomes in preeclampsia pregnant women by ultrasonic images on account of intelligent algorithms. The automatic labeling of fetal cerebral artery in fetal MCA hemodynamic images was achieved through the HSegNet algorithm model, and it had a good consistency with the manual labeling by doctors. Education was a protective factor for preeclampsia. BMI and family history of hypertension were independent risk factors for preeclampsia. However, there are still some limitations in the study. The number of research samples in the study is not large enough, and the indicators are not comprehensive enough. Therefore, it is necessary to increase the number of samples and combine multiple indicators to comprehensively and objectively evaluate the blood circulation and intrauterine status of the preeclampsia fetuses. In conclusion, the intelligent algorithm-based fetal MCA hemodynamic ultrasound image in this study can effectively predict the pregnancy outcomes of patients and provide certain theoretical supports for the subsequent reduction of adverse pregnancy outcomes in patients with preeclampsia.

## Data Availability

The data used to support the findings of this study are available from the corresponding author upon request.
